# Cytokines secreted from adipose tissues mediate tumor proliferation and metastasis in triple negative breast cancer

**DOI:** 10.1186/s12885-022-09959-6

**Published:** 2022-08-13

**Authors:** Kai Zhang, Lin Chen, Hongbo Zheng, Yi Zeng

**Affiliations:** 1 Head and Neck Breast Surgery, The Yuebei People’s Hospital of Shaoguan, Guangdong Province 512025 Shaoguan, China; 2 Department of Anesthesiology, Maternal and Child Health Hospital of Hubei Province, Hubei Province 430070 Wuhan, China; 3 Department of Medicine, Genecast Biotechnology Co., Ltd, Jiangsu Province 214000 Wuxi, China; 4 Clinical Oncology School of Fujian Medical University, Fujian Cancer Hospital, No. 420 Fuma Road, Fuzhou, Fujian Province 350014 China

**Keywords:** Triple-negative breast cancer, Obesity, Cytokines, Proliferation, Migration

## Abstract

**Background:**

Obesity is a high-risk factor for development and poor prognosis of triple-negative breast cancer (TNBC), which was considered as a high malignant and poor clinical outcome breast cancer subtype. TNBC proliferation and migration regulated by obesity is complex. Here, we studied effects of cytokines secreted from adipose tissue on development of TNBC.

**Methods:**

Forty postmenopausal cases by Yuebei People’s Hospital of Shaoguan with stage I/IIA TNBC were enrolled. Cytokine concentrations were examined using ELISA analysis. Proliferation and migration of TNBC cell lines were performed using CCK8 assays and Transwell tests. The Log-rank (Mantel-Cox) test, two-tailed Mann-Whitney U test and two-tailed unpaired *t* test were performed using GraphPad Prism 8.4.2.

**Results:**

Survival analysis indicated that obese patients with TNBC had worse disease free survival (DFS) as compared with normal weight group (Hazard Ratio 4.393, 95% confidence interval (CI) of ratio 1.071–18.02, *p* < 0.05). Obese patients with TNBC had severe insulin resistance and high plasma triglycerides. However, plasma adiponectin concentration was decreased and interleukin-6 (IL-6) and tumor necrosis factor-α (TNF-α) concentration was increased in obese TNBC patients as compared with the nonobese group. The similar results were found in the cytokine secretion from adipose tissues and insulin-resistant adipocytes. The secretion of adipose tissue from obese TNBC patients could promote proliferation and migration of TNBC cell lines, including MDA-MB-157, MDA-MB-231, MDA-MB-453 and HCC38 cells. These TNBC cell lines co-incubated with insulin-resistant 3T3-L1 adipocytes or supplementing these cytokines medium also exhibited increase of proliferative and migratory capacity.

**Conclusion:**

TNBC patients with obesity had worse prognosis compared with the normal weight groups. Alteration of cytokines secreted from adipose tissues mediated proliferation and migration of TNBC, leading to tumor progression in TNBC patients with obesity.

**Supplementary Information:**

The online version contains supplementary material available at 10.1186/s12885-022-09959-6.

## Background

Female breast cancer has surpassed lung cancer as the most commonly diagnosed cancer, estimated by GLOBOCAN 2020 [1]. Although mortality of breast cancer is only 6.9%, the large population base diagnosed with breast cancer (2.3 million new cases in 2020) has to gained attention from scientists and specialists [1]. Adipose tissue is one of the compositions of breast and has an important influence on occurrence and development of breast cancer [2]. As the global epidemic of obesity, obesity-associated breast cancer patients are increasingly diagnosed each year [1–3]. Obese breast cancer patients also have higher risk of aggressive tumor phenotype and therapy resistance [4]. Higher body mass index (BMI) is significantly associated with an in increased risk of aggressiveness among breast cancer patients [5, 6]. Many studies indicate that obesity decreases therapeutic outcome for breast cancer, including chemotherapy [7], radiotherapy [8] and endocrine therapy [9].

Triple-negative breast cancer (TNBC) was considered as a high malignant and poor clinical outcome breast cancer subtype. The lack of estrogen receptor (ER) and progesterone receptor (PR) expression and epidermal growth factor receptor 2 (HER2/ERBB2) amplification and/or overexpression is a common characteristic of TNBC [10]. TNBC patients exhibited aggressive pathology and worse disease free survival (DFS) and overall survival (OS) and were significantly younger compared with breast cancer patients with non-TNBC [11]. Chemotherapy remains a main strategy for TNBC treatment [11, 12], although many new treatment strategies, such as immunotherapy, target therapy and neoadjuvant treatments, were explored and studied [13–15].

Obesity is an important risk factor for TNBC outcome and many clinical trials indicate that obesity leads to shorter DFS and OS compared with TNBC patients of normal body weight [3, 16, 17]. However, the mechanistic link between TNBC and obesity is still unclear in the previous studies. In the present study, we explored effects of obesity on TNBC patient outcomes. We evaluated insulin sensitivity and change of cytokines including adiponectin, interleukin-6 (IL-6) and tumor necrosis factor-α (TNF-α) in obesity and normal body weight TNBC patients, studied influence of cytokines secreted by insulin resistant adipocytes to tumor proliferation and migration in TNBC cell lines.

## Materials and methods

### Patients and clinical data

The present study enrolled 40 postmenopausal cases by Yuebei People’s Hospital of Shaoguan with stage I/IIA triple-negative (ER and/or PR were none or weak expression (less than 10% cells stained by immunohistochemistry (IHC)); HER2 was negative (0–1+) using IHC) breast cancer from May 2014 to April 2016. BMI (kg/m^2^) of patients was evaluated before surgery. The studies were reviewed and approved by the Ethics Committee of Yuebei People’s Hospital of Shaoguan, and treatment and extended follow-up data were retrospectively assembled with medical ethics committee approval. All experiments were performed in accordance with relevant guidelines and regulations. The TNBC patients was 47–70 years old, and median age was 58 years old. The nonobese group was 20 cases and BMI was 18.5–22.8 kg/m^2^; the obese group was 20 cases and BMI was 29.5–38.7 kg/m^2^. These patients included 10 cases with type 2 diabetes, 7 cases of which were contained in the obese group. The detailed clinical characteristics were summarized in Table 1.

**Table 1:** Distribution of clinical characteristics by the nonobese/obese groups

Category	Total (n = 40)	Nonobese (n = 20)	Obese (n = 20)
Age (Median/Range)	58 (47–70)	59 (51–68)	56.5 (47–70)
Menopausal status (n/%)			
Postmenopausal	40 (100%)	20 (100%)	20 (100%)
Tumor stage (n/%)			
I	17 (42.5%)	8 (40%)	9 (45%)
IIA	23 (57.5%)	12 (60%)	11 (55%)
Molecular subtype (n/%)			
Triple negative	40 (100%)	20 (100%)	20 (100%)
BMI (Median/Range)	26.15 (18.5–38.7)	20.9 (18.5–22.8)	32.55 (29.5–38.7)
Type 2 diabetes mellitus (n/%)			
YES	10 (25%)	3 (15%)	7 (35%)
NO	30 (75%)	17 (85%)	13 (65%)

### Cell culture

TBNC cell lines, including MDA-MB-157, MDA-MB-231, MDA-MB-453 and HCC38 cells, were purchased from the Cell Bank of Type Culture Collection of the Chinese Academy of Sciences (Shanghai, China). 3T3-L1 preadipocytes were purchased from the American Type Culture Collection (ATCC, Manassas, USA). MDA-MB-157 and MDA-MB-453 cells were cultured in Leibovitz’s L-15 Medium containing 10% fetal bovine serum (FBS, Gibco, Grand Island, USA). MDA-MB-231 cells were cultured in DMEM (Gibco, Grand Island, USA) containing 10% FBS and HCC38 was cultured in PRMI Medium (Gibco, Grand Island, USA) containing 10% FBS. 3T3-L1 preadipocytes were cultured in DMEM/F12 (Gibco, Grand Island, USA) containing 10% FBS. These cells were cultured in a 5% CO_2_ and 37 °C humidified incubator.

### Insulin-resistant 3T3-L1 adipocyte model

3T3-L1 preadipocytes were induced and differentiated into 3T3-L1 adipocytes [18]. 3T3-L1 preadipocytes were cultured using 3T3-L1 differentiation kit (Sigma-Aldrich, USA). Differentiation 3T3-L1 adipocytes were conformed using oil red O staining. For insulin-resistant 3T3-L1 adipocyte model, 3T3-L1 adipocytes were cultured 10% FBS DMEM/F12 medium supplemented with 20 nM Dexamethasone for 6 days [19]. The model was confirmed using glucose absorption method (Glucose Oxidase Method (GOM) assay kit, Applygen Technologies Inc., China) after 10 nM insulin treatment for 1 h.

### Cytokine detection

Adiponectin, IL-6 and TNF-α examinations of plasma and tissue secretion were used by Adiponectin (human) ELISA Assay Kit (BioVision, USA), IL-6 (human) ELISA Kit (BioVision, USA) and Human TNF-α ELISA Kit (Abbkine, USA) according to the manufacturers’ instructions, respectively. Adipose tissues isolated from fresh surgery samples of TNBC cases were incubated in culture medium for 12 h and the culture medium was collected and measured. Adiponectin, IL-6 and TNF-α examinations of culture medium incubated with normal and insulin-resistant 3T3-L1 adipocytes were used by Adiponectin (mouse) ELISA Assay Kit (BioVision, USA), IL-6 (mouse) ELISA Kit (BioVision, USA) and Mouse TNF-α ELISA Kit (Abbkine, USA) according to the manufacturers’ instructions, respectively. Normal and insulin-resistant 3T3-L1 adipocytes were incubated in fresh culture medium for 24 h and the culture medium were collected and detected.

### Cell proliferation and migration

Cell proliferation was measured using the Cell Counting Kit-8 (CCK8, Dojindo, Japan). MDA-MB-157, MDA-MB-231, MDA-MB-453 or HCC38 cells were cultured in 96-well plates at a density of 5 × 10^3^ cells/well for 24 h, then treated with adipose secretion for 24 h. Subsequently, CCK8 test was performed and absorption were measured at 490 nm light. For cell proliferation in co-culture TNBC cell line and adipocytes, TNBC cell lines were seeded in Transwell filter of Transwell culture plates (Corning Costar, USA), and normal/insulin-resistant 3T3-L1 adipocytes were seeded in the low well. After 24 h, the TNBC cells in the Transwell culture plates were collected, transferred to 96-well plates and measured using CCK8.

Cell migration was examined using the Transwell culture. Four TNBC cell lines were seeded in Transwell filters at a density of 5 × 10^5^ cells/well for 12 h, and adipose secretion or 3T3-L1 adipocytes were placed in the low well. After 24 h, the nonmigrating TNBC cells in the upper surface of Transwell filters were removed and the migrating TNBC cells in the lower surface were collected and transferred to 96-well plates and examined using CCK8.

### Statistical analysis

Statistical analysis was performed using GraphPad Prism 8.4.2. Survival analysis was Kaplan-Meier analysis using the Log-rank (Mantel-Cox) test. Metabolic indexes and cytokines analysis was the two-tailed Mann-Whitney U test. Cells and tissues tests were analyzed using the two-tailed unpaired *t* test. *p* < 0.05 was considered to a statistically significant difference.

## Results

### Obesity decreased survival of TNBC patients

**Fig. 1:**
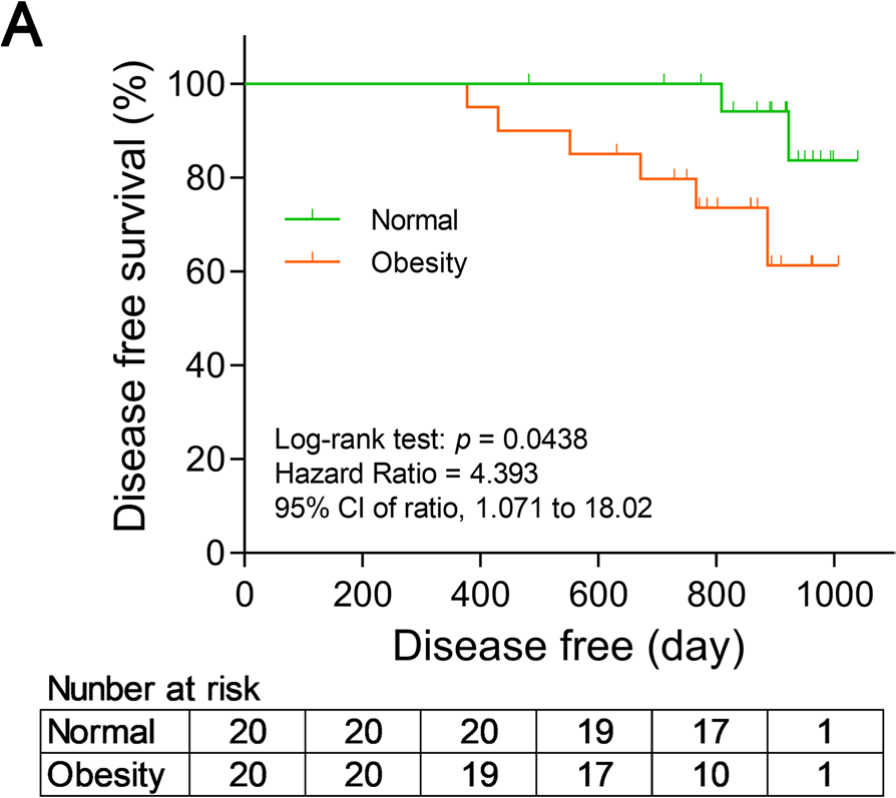
Obesity reduced disease free survival in triple-negative breast cancer (TNBC) patients. Kaplan-Meier analysis of disease free survival (DFS) according to body mass index (BMI). Normal weight group, BMI (18.5–22.8), *n* = 20; obese group, BMI (29.5–38.7), *n* = 20. Statistics based on the log-rank (Mantel-cox) test

The clinicopathological characteristics of 40 postmenopausal patients with TNBC were summarized in Table 1. We screened the TNBC patients which were divided into nonobese (Median/Range: 20.9 kg/m^2^/18.5–22.8 kg/m^2^) and obese (Median/Range: 32.55 kg/m^2^/29.5–38.7 kg/m^2^) group according to BMI. Median (Range) in the nonobese and obese groups was 59 (51–68) and 56.5 (47–70), respectively. Twelve (60%) cases in the nonobese group and 11 (55%) cases in the obese group were stage IIA, the remaining cases were stage I TNBC. These cases contained 7 (35%) patients with type 2 diabetes in the obese group and 3 (15%) patients with type 2 diabetes in the nonobese group. Through 3 years of follow-up, we found that DFS was significantly different (*p* = 0.0438; Hazard ratio = 4.393; 95% CI of ratio: 1.071–18.02) between the nonobese and obese cohorts, revealed that obese patients with TNBC had a worse survival (Fig. 1 A). Thus, excessive adipose tissue promotes progression of tumor and affects proliferation and migration of TNBC.

### Obesity caused blood parameter changes in TNBC

**Fig. 2:**
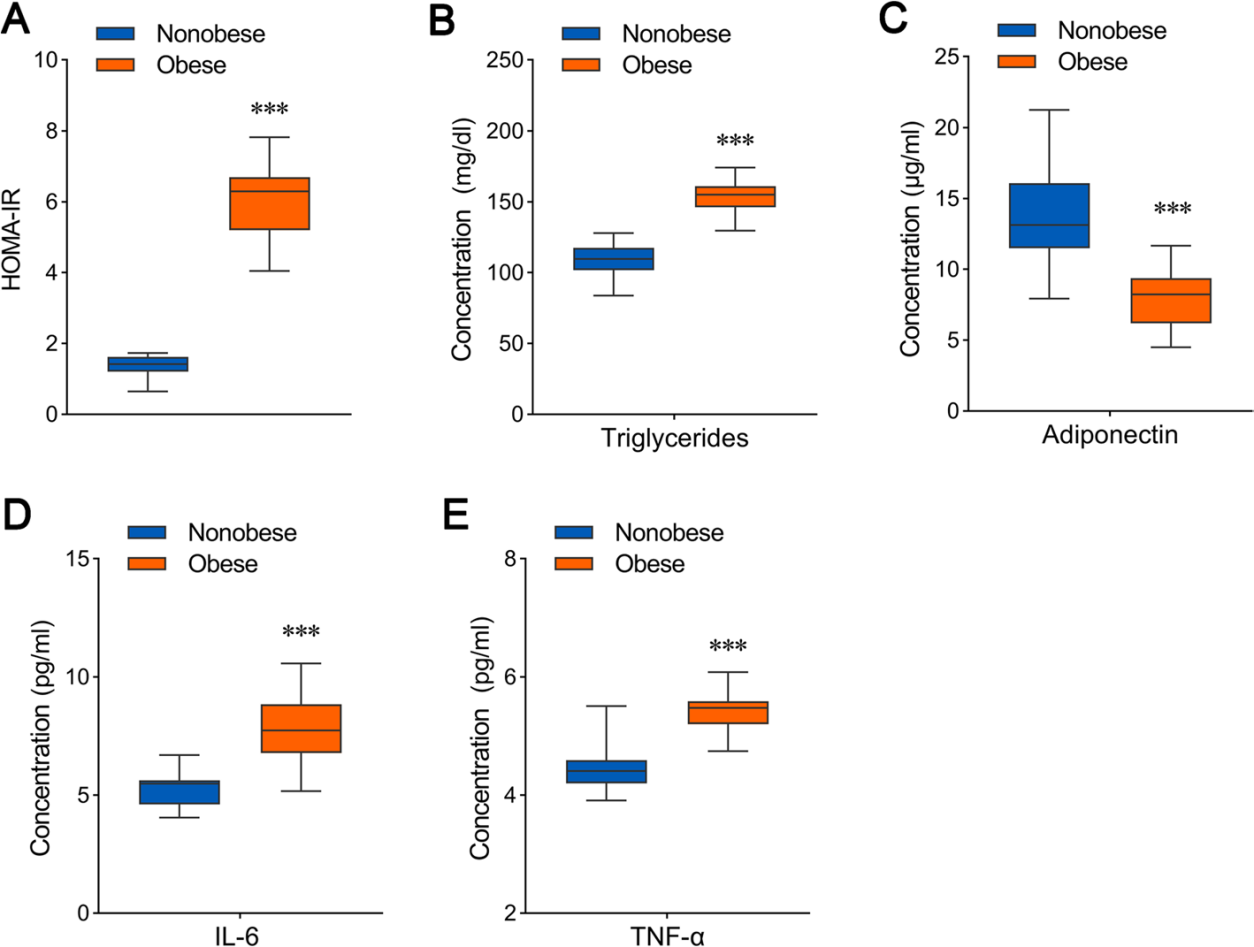
Difference of metabolic indexes and cytokines between nonobese and obese groups in TNBC patients. Difference analysis of the homeostasis model assessment-estimated insulin resistance (HOMA-IR) index (**A**), and concentration of serum triglycerides (**B**), plasma adiponectin (**C**), plasma interleukin-6 (IL-6) (**D**) and plasma tumor necrosis factor-α (TNF-α) (E) between nonobese group (*n* = 20) and obese group (*n* = 20) in TNBC patients. Statistics based on the two-tailed Mann-Whitney U test. ***p < 0.001

It is well known that insulin resistance is a common characteristic in obesity and type 2 diabetes and blood glucose and blood lipid are higher compared with healthy people. We evaluated alteration of insulin resistance and serum triglycerides between nonobese and obese patients with TNBC. As shown in Fig. 2 A, severe insulin resistance was observed in the obese TNBC cohort using analysis of homeostasis model assessment-estimated insulin resistance (HOMA-IR). Meanwhile, serum triglycerides levels were higher in the obese group compared with the nonobese group (Fig. 2B). We further examined levels of plasma cytokines including adiponectin, IL-6 and TNF-α in the TNBC patients. Concentration of adiponectin was decreased in the obese cohort with TNBC (Fig. 2 C), however, concentration of IL-6 and TNF-α was increased in the obese group compared with the nonobese group (Fig. 2D and E). These results indicated that obesity did not only change metabolic features but also levels of plasma cytokines in TNBC patients.

## Obesity altered cytokine secretion of adipose tissue

**Fig. 3:**
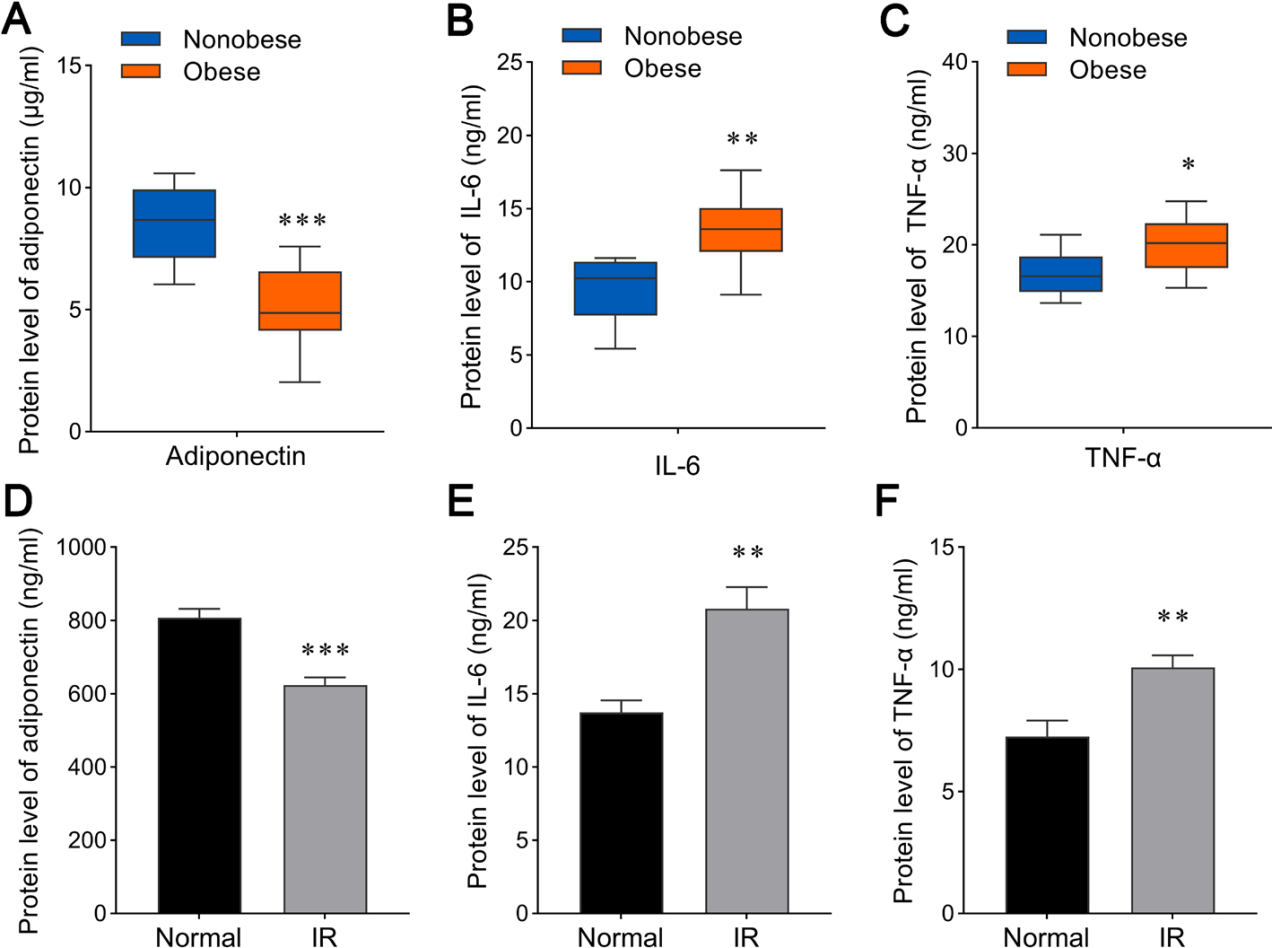
Cytokine secretion from adipose tissues and cells. **A**-**C** Secretion of adiponectin (**A**), IL-6 (**B**) and TNF-α (**C**) from adipose tissues in nonobese and obese TNBC patients. *n* = 10. Statistics based on the two-tailed Mann-Whitney U test. *p < 0.05, **p < 0.01, ***p < 0.001. (**D**-**F**) Secretion of adiponectin (**D**), IL-6 (E) and TNF-α (**F**) from normal and insulin-resistant (IR) 3T3-L1 adipocytes. *n* = 5. Statistics based on the unpaired *t* test. **p < 0.01, ***p < 0.001

Adipose tissue is one of main sources of adiponectin, IL-6 and TNF-α. Obesity caused alteration of plasma adiponectin, IL-6 and TNF-α in TNBC patients. To explore reasons of the changes, we examined three cytokine levels secreted from adipose tissue isolated from surgery samples of TNBC cases. Variation tendency of three cytokines was consistent with the results of plasma cytokines (Fig. 3 A-C). Adiponectin secretion from adipose tissue was reduced in the obese TNBC samples (Fig. 3 A). Levels of IL-6 and TNF-α in the medium incubated with adipose tissue from the obese TNBC samples were enhanced compared with the nonobese TNBC samples (Fig. 3B and C).

We further constructed an insulin-resistant 3T3-L1 adipocyte model. 3T3-L1 adipocytes were stained by red oil O through cell differentiation for 8 days (Fig. S1A). Effect of glucose absorption in insulin-resistant 3T3-L1 adipocytes was significantly reduced after insulin treatment compared with normal 3T3-L1 adipocytes (Fig. S1B). In the adipocytes model, we also found that adiponectin (Fig. 3D) secreted by insulin-resistant 3T3-L1 adipocytes was decreased and IL-6 (Fig. 3E) and TNF-α (Fig. 3 F) secreted by insulin-resistant 3T3-L1 adipocytes was increased as compared with normal 3T3-L1 adipocytes. These results revealed that insulin resistance of adipose tissues and cells caused alteration of cytokine secretion.

### Secretion from insulin-resistant adipose tissues promoted proliferation and migration of TNBC

**Fig. 4:**
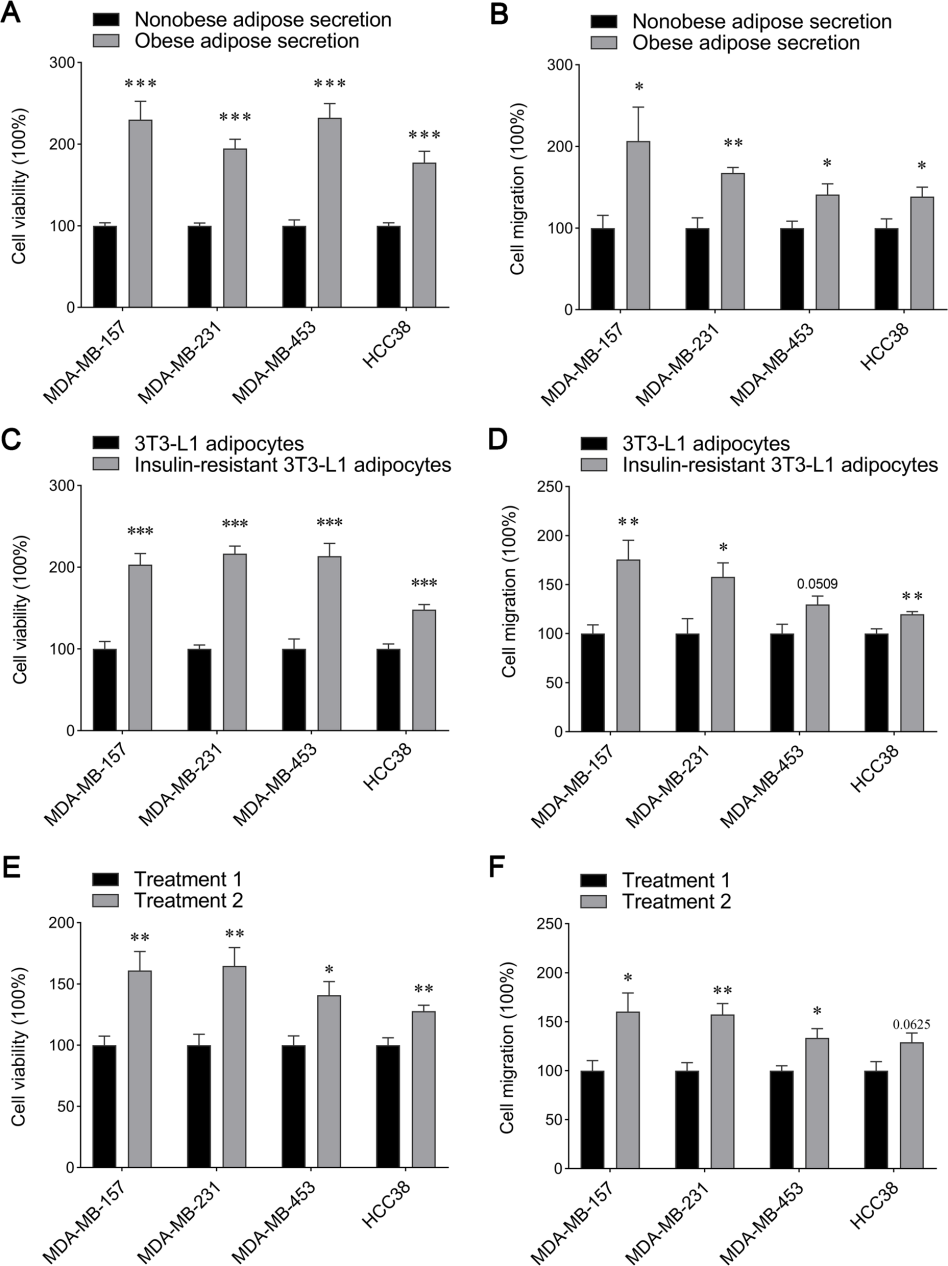
Proliferation and migration effects of cytokines secreted by adipose tissues and cells on TNBC cell lines. **A** Cell viability analysis was detected in MDA-MB-157, MDA-MB-231, MDA-MB-453 and HCC38 cells incubated with adipose secretion from nonobese and obese TNBC patients for 24 h. *n* = 5. Statistics based on the unpaired *t* test. ***p < 0.001. **B** Migration of MDA-MB-157, MDA-MB-231, MDA-MB-453 and HCC38 cells treated with adipose secretion from nonobese and obese TNBC patients for 24 h were detected using a Transwell coculture method. *n* = 5. Statistics based on the unpaired *t* test. *p < 0.05, **p < 0.01. **C**-**D** Proliferation (**C**) and migration (**D**) of MDA-MB-157, MDA-MB-231, MDA-MB-453 and HCC38 cells co-cultured with normal or insulin-resistant 3T3-L1 adipocytes were examined using a Transwell coculture method. *n* = 5. Statistics based on the unpaired *t* test. *p < 0.05, **p < 0.01, ***p < 0.001. (E-F) Proliferation (**E**) and migration (**F**) of different TNBC cell lines with different treatments. Treatment 1 was the cells co-incubated with 9 µg/ml adiponectin, 10 ng/ml IL-6 and 17 ng/ml TNF-α; treatment 2 was the cells co-incubated with 5 µg/ml adiponectin, 14 ng/ml IL-6 and 20 ng/ml TNF-α. *n* = 5. Statistics based on the unpaired *t* test. *p < 0.05, **p < 0.01

To confirm proliferation and migration effects of insulin-resistant adipose tissues on TNBC, we cultured four TNBC cell lines, including MDA-MB-157, MDA-MB-231, MDA-MB-453 and HCC38 cells. Four TNBC cell lines were co-cultured with secretion of adipose tissues from TNBC surgery samples. The results indicated that viability of MDA-MB-157, MDA-MB-231, MDA-MB-453 and HCC38 cells co-cultured with secretion of adipose tissues from obese TNBC samples was significantly enhanced as compared with the nonobese TNBC sample group (Fig. 4A). Cell migration assay using Transwell culture revealed that adipose tissue secretion in the obese TNBC group promoted migration of TNBC cell lines (Fig. 4B). The similar results were observed in TNBC cell lines co-cultured with normal or insulin-resistant 3T3-L1 adipocytes (Fig. 4C and D). These data demonstrated that secretion from insulin-resistant adipose tissues and cells promoted proliferation and migration of TNBC cells, which might be an important factor to cause low outcome and survival in obese TNBC cases.

To further explore roles of adiponectin, IL-6 and TNF-α in proliferation and migration of TNBC cells, we supplemented with adiponectin, IL-6 and TNF-α in culture medium and studied behavior of TNBC cell lines. The supplemental concentration of adiponectin, IL-6 and TNF-α was determined according to release protein levels of adipose tissues in Fig. 3 A-C. Proliferation ability of MDA-MB-157, MDA-MB-231, MDA-MB-453 and HCC38 cells supplemented with low concentration of adiponectin and high concentration of IL-6 and TNF-α was significantly increased (Fig. 4E). Meanwhile, migration ability was also enhanced in MDA-MB-157, MDA-MB-231 and MDA-MB-453 cells supplemented with low concentration of adiponectin and high concentration of IL-6 and TNF-α compared with the control group (Fig. 4 F). Although only three cytokines secreted by adipose tissues and cells were studied, our results demonstrated that it was important roles of adiponectin, IL-6 and TNF-α to promote proliferation and migration of TNBC.

## Discussion

Previous many studies explored effects of obesity on breast cancer outcome, such as the SUCCESS A trial [3, 20], the BIG 02–98 trial [21], meta-analysis [4, 22]. Some researches indicated that there was shorter DFS and OS in obese breast cancer patients (BMI ≥ 30 kg/m^2^) compared with nonobese patients (BMI < 30 kg/m^2^) [21, 23]. However, results from the randomized SUCCESS A trial with 3754 breast cancer patients indicated that there was no significant different between obese (BMI ≥ 30 kg/m^2^) and nonobese (BMI < 30 kg/m^2^) breast cancer patients, and severe obese patients (BMI ≥ 40.0 kg/m^2^) had a worse DFS and OS compared nonobese patients (BMI < 25 kg/m^2^) [3]. More and more demonstrations indicate that BMI may become an independent prognostic factor for DFS and OS in TNBC although the effect of BMI is controversial in all breast cancer subtypes [3, 4, 16, 17]. In the present study, we also revealed that obese (29.5–38.7 kg/m^2^) patients with TNBC were shorter DFS compared with the normal weight (18.5–22.8 kg/m^2^) patients (HR 4.393, 95%CI 1.071–18.02, *p* < 0.05). These evidences reveal that exorbitant BMI leads to worse prognosis in early TNBC patients.

Obesity is an important risk factor for metabolic syndrome, type 2 diabetes, nonalcoholic fatty liver disease [24]. Previous study indicated that metabolic syndrome caused increased incidence of TNBC [25]. Insulin resistance is closely associated with obesity and type 2 diabetes [26, 27]. Recent study also showed that insulin resistance led to poor prognosis in breast cancer patients [28]. Our data indicated that obese patients had severe insulin resistance and abnormalities of glucose-lipid metabolism. Meanwhile, these obese TNBC patients had worse prognosis compared with the normal weight group. These data suggested that metabolic disorder was involved in progression of TNBC. We also found that proliferation and migration of TNBC cell lines (including MDA-MB-157, MDA-MB-231, MDA-MB-453 and HCC38 cells) were enhanced co-incubated with insulin-resistant 3T3-L1 adipocytes. Thus, insulin-resistant adipose tissue is primary reasons of poor outcome in obese TNBC patients.

Adipose tissue, as everyone knows, is a mainly energy storage organ. It is also an important endocrine organ [29, 30]. So far over 100 kinds of adipocytokines are found, such as adiponectin, TNF-α, IL-6, leptin and so on [31–33]. Metabolic abnormality of adipose tissue not only affects glucose-lipid regulation, but also changes cytokine secretion. Adiponectin is an antimitogenic and anti-angiogenic hormone, which mainly is secreted from maturation adipocytes, but promitogenic leptin is low levels from maturation adipocytes [34]. Adiponectin is involved in inhibition of cell proliferation and angiogenesis [35]. Adiponectin expression in adipose tissues from obese patients is lower than from nonobese people [36]. Low expression of adiponectin in adipose tissue relieves inhibition of TNBC development. TNF-α and IL-6, as proinflammatory factors, from obese adipose tissue contribute to insulin resistance by impairing insulin receptor activation, upregulating insulin and insulin-like growth factor-1 (IGF-1) levels [37]. IGF-1 receptor is overexpressed in TNBC and promotes breast cancer growth [38]. Basal concentrations of TNF-α and IL-6 in obese patients are increase and the high levels are reduced with weight loss [39]. Thus, obese adipose tissue provides a suitable environment for TNBC proliferation and migration. Our data indicated that secretion of adipose tissues from obesity with TNBC and co-incubation with insulin-resistant 3T3-L1 adipocytes promoted proliferation and migration of TNBC cell lines. Consistent with these results, different supplementation with adiponectin, TNF-α and IL-6 changed development of TNBC cells. These demonstrations indicate that levels of adiponectin, TNF-α and IL-6 from adipose tissue affect TNBC progression, although we cannot exclude effects of another cytokines from adipose tissue.

**Fig. 5:**
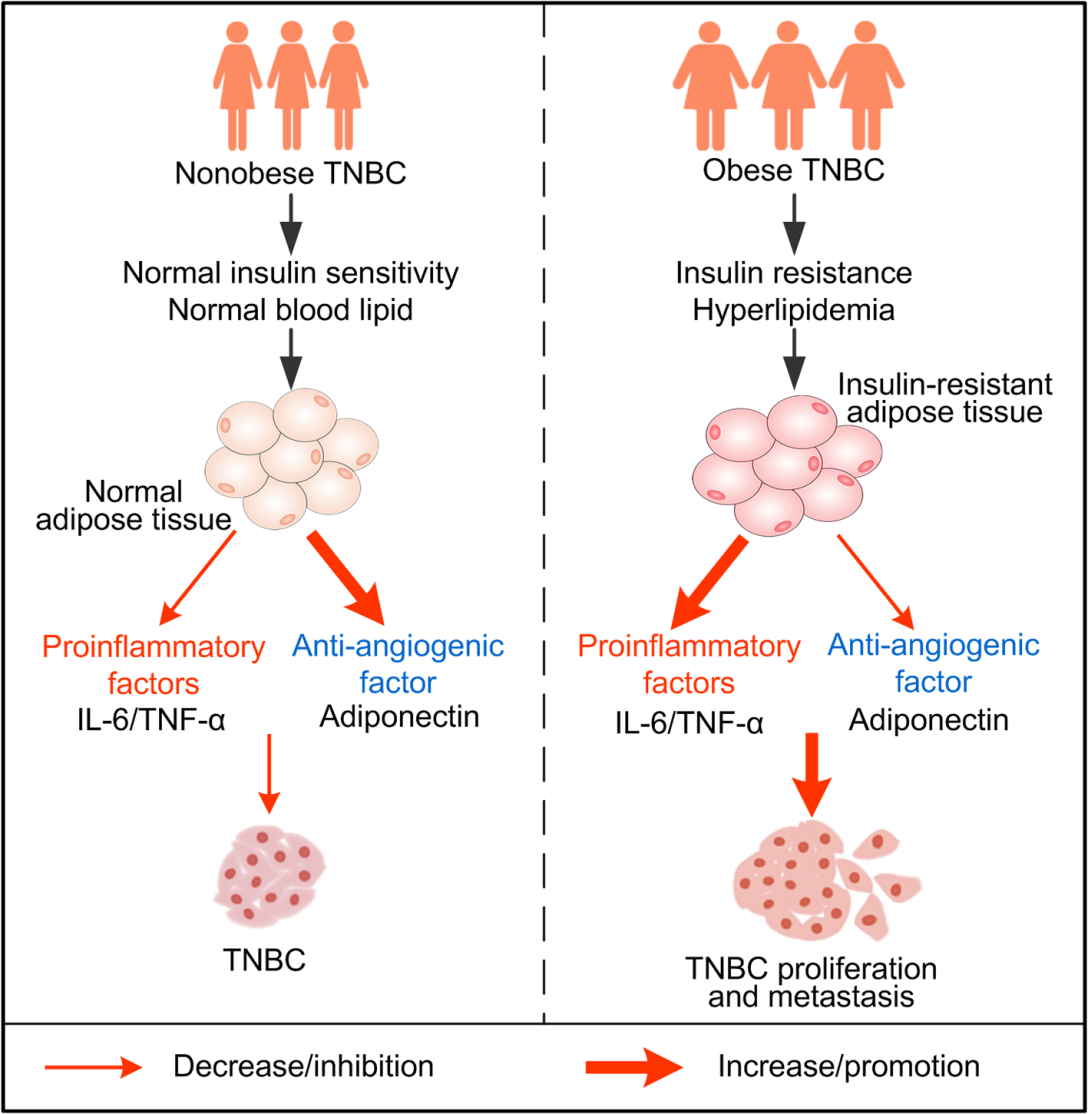
Regulation mechanism of TNBC proliferation and migration through cytokines secreted from adipose tissue in nonobese and obese patients

The present study revealed that TNBC patients with obesity had shorter DFS compared with normal weight group. These obese TNBC patients also had abnormal glucose-lipid metabolism and severe insulin resistance. Cytokines (adiponectin, TNF-α and IL-6) secreted from adipose tissues of obese TNBC patients and insulin-resistant 3T3-L1 adipocytes were also abnormal (Fig. 5). The abnormal cytokine levels promoted TNBC cells proliferation and migration, suggesting that insulin-resistant adipose tissue affects TNBC progression mediated by cytokines.

## Conclusion

Excessive obesity causes worse prognosis of TNBC patients. Obesity induces insulin resistance and glucose-lipid metabolism, altering cytokines secreted from adipose tissue. The change of cytokine secretion promotes proliferation and migration of TNBC, which causes tumor progression in TNBC patients with obesity.

## Supplementary Information

**Additional file 1:** **Figure S1.** Insulin-resistant model of 3T3-L1 adipocytes.

## Data Availability

The raw data supporting the conclusions of this article will be made available by Kai Zhang (zkkkk1@yeah.net), Lin Chen (Lin_Chen17@163.com) and Yi Zeng (zengyifc@126.com), without undue reservation.

## Abbreviations

BMI: Body mass index
TNBC: Triple-negative breast cancer
ER: Estrogen receptor
PR: Progesterone receptor
HER2/ERBB2: Epidermal growth factor receptor 2
DFS: Disease free survival
OS: Overall survival
IL-6: Interleukin-6
TNF-α: Tumor necrosis factor-α
IHC: Immunohistochemistry
FBS: Fetal bovine serum
HOMA-IR: Homeostasis model assessment-estimated insulin resistance
