# Supplementary material for: Cytokines secreted from adipose tissues mediate tumor proliferation and metastasis in triple negative breast cancer
Source: BMC Cancer. 2022 Aug 13;22:886. doi: 10.1186/s12885-022-09959-6 (PMC9375239; doi:10.1186/s12885-022-09959-6)
Supplement: Supplementary file 1 — Additional file 1: Figure S1. Insulin-resistant model of 3T3-L1 adipocytes. [file 12885_2022_9959_MOESM1_ESM.docx]

**
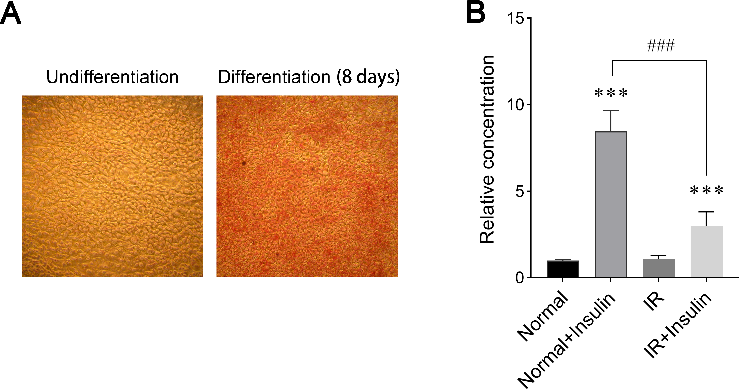
**

**Figure S1. Insulin-resistant model of 3T3-L1 adipocytes.** (A) Representative red oil O staining of 3T3-L1 preadipocytes and adipocytes. 3T3-L1 preadipocyte differentiated into 3T3-L1 adipocytes incubated with the differentiation medium for 8 days. (B) Glucose absorption by 3T3-L1 adipocytes and insulin-resistant 3T3-L1 adipocytes 1 h after 10 nM. Statistics based on the unpaired t test. ****p* < 0.001, ###*p* < 0.001.


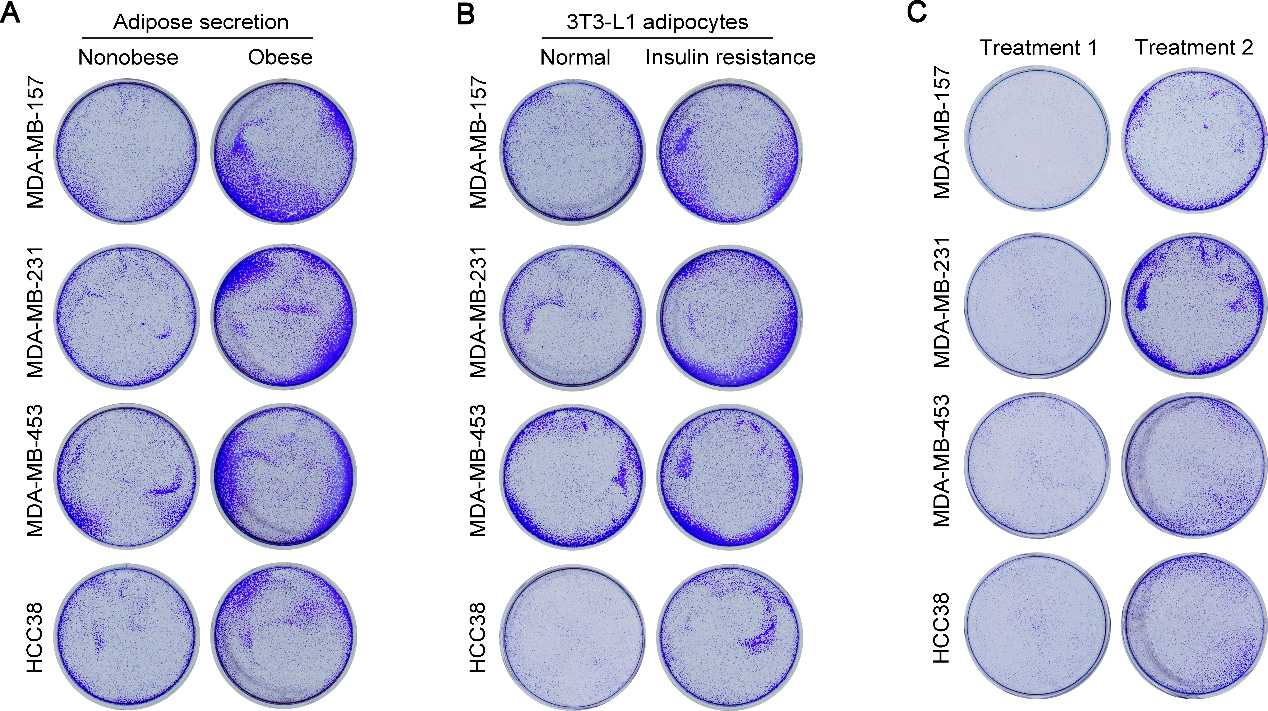


**Figure S2. Migration of TNBC cell lines treated with cytokines secreted by adipose tissues and cells.** (A-C) MDA-MB-157, MDA-MB-231, MDA-MB-453 and HCC38 cells were co-cultured with adipose secretion (A), 3T3-L1 adipocytes (B), or different concentration of adiponectin, IL-6 and TNF-α (C) for 24 h using 12 well Transwell plates. The detail treatments were consistent with Figure 4B, 4D and 4F. The colony formation was stained with crystal violet staining solution and imaged.
